# Pseudolipoblastic Perineuroma: A Rare Histologi̇c Subtype

**DOI:** 10.1097/DAD.0000000000002857

**Published:** 2024-10-15

**Authors:** Ozlem Erdem, Barbara Corti, Francesco Paolo Salamone, Bianca Maria Piraccini, Cosimo Misciali

**Affiliations:** * Department of Pathology, Faculty of Medicine, Gazi University, Besevler/Ankara, Turkiye;; † Referente Percorsi Di Patologia Cutanea-DIAP, Un Anatomia Patologica, IRCCS Aoubo-Policlinico Di'S'Orsola, Via Massarenti, Bologna, Italy;; ‡ U.O.C. Dermatologia, IRCCS Azienda Ospedaliero-Universitaria di Bologna Italia, Bologna, Italy; and; § Dipartimento di Scienze Mediche e Chirurgiche Università di Bologna Italia, Via Massarenti, Bologna, Italy.

**Keywords:** perineurioma, pseudolipoblastic, immunohistochemistry, soft tissue tumors

## Abstract

Pseudolipoblastic perineurioma is a very uncommon variant of extraneural perineurioma, with only a limited number of cases documented in the medical literature. The most remarkable histopathologic characteristic is the existence of vacuolated cells that closely resemble lipoblasts; besides the presence of small, spindle shaped, or epithelioid perineurial cells. In this study, we present another case of pseudolipoblastic perineurioma, predominantly characterized by the presence of vacuolated “pseudolipoblastic” cells. The immunohistochemical expression of EMA, Glut-1, claudin-1, collagen type IV, and laminin as well as S-100 negativity is essential for the diagnosis to support the perineurial origin. Simple excision is the best treatment option for these benign tumors that do not recur or metastasize. It is crucial to recognize this rare entity to differentiate it from many other tumors characterized by prominent intracytoplasmic vacuoles.

## INTRODUCTION

Perineuriomas are rare, benign tumors of the peripheral nerve sheath, composed of perineurial cells. Clinical presentation of this entity is as solitary, painless, skin-colored, well-defined papules or nodules.^1^ They occur slightly more commonly in middle-aged women with a wide distribution.^2^ These tumors are classified based on their relationship with the histologic boundaries of the nerve from which they originate as intraneural and extraneural perineuriomas.^3^

Although they are rare, many morphologic variants have been described in the literature, such as sclerosing,^4^ sclerosing pacinian-like,^5^ reticular/microcystic,^6^ epithelioid,^7^ granular cell,^8^ cutaneous fibrous,^9^ plexiform,^10^ tendon sheath fibroma-like,^11^ pseudolipoblastic,^12^ and microreticular perineurioma.^13^ In addition to these rare morphologic variants, perineuriomas can be seen as a component of hybrid tumors such as hybrid schwannoma/perineurioma,^14,15^ hybrid perineurioma/neurofibroma,^15^ and hybrid perineurioma/neurothekeoma.^16^

Herein, we describe a case of pseudolipoblastic perineurioma in a 42-year-old woman on the right hand.

## CASE REPORT

A 42-year-old woman presented with a nodular lesion on the right thumb. The patient was asymptomatic and she had no history of trauma. Her family and personal history were unremarkable for a specific disease (such as fibromatosis). The lesion was surgically excised with a clinical diagnosis of myopericytoma.

## MATERIALS AND METHODS

After the fixation in formaldehyde and processed routinely, the tissue is embedded in paraffin. Pathologic slides were stained with hematoxylin and eosin. Immunohistochemistry was also performed on the BenchMark ULTRA automated immunostainer (Ventana Medical Systems- Roche Diagnostics, Switzerland) with antibodies against vimentin, EMA, Glut-1, CD34, Smooth Muscle Actin (SMA), S-100, CD31, Calponin, Desmin, CK AE1/AE3, p63, Sox10, HMB-45, neurofilament, ERG, and calretinin.

## RESULTS

**FIGURE 1.**
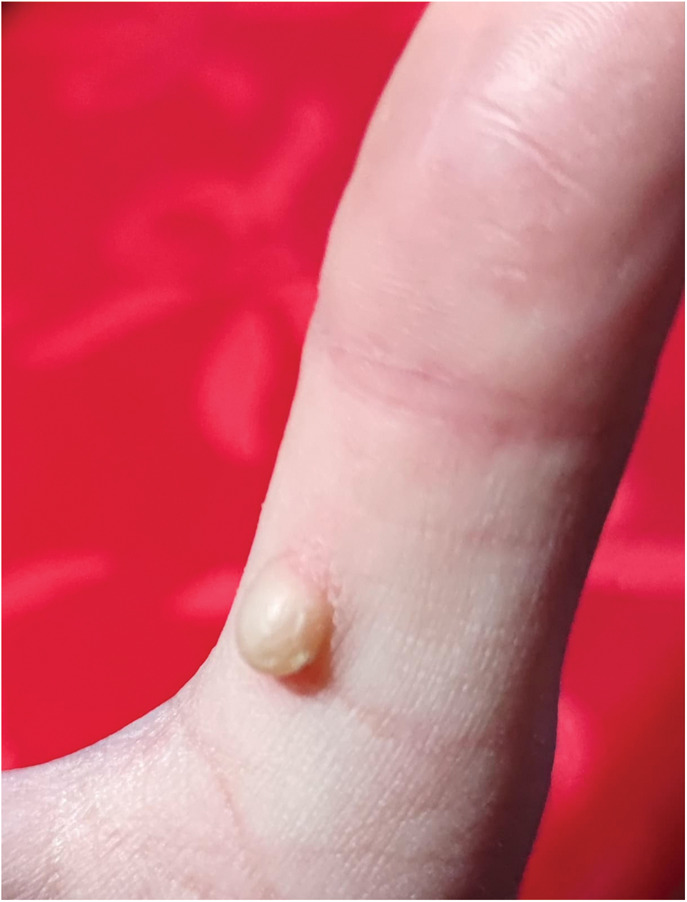
Clinical photograph of the nodular well-demarcated lesion on right thumb.

**FIGURE 2.**
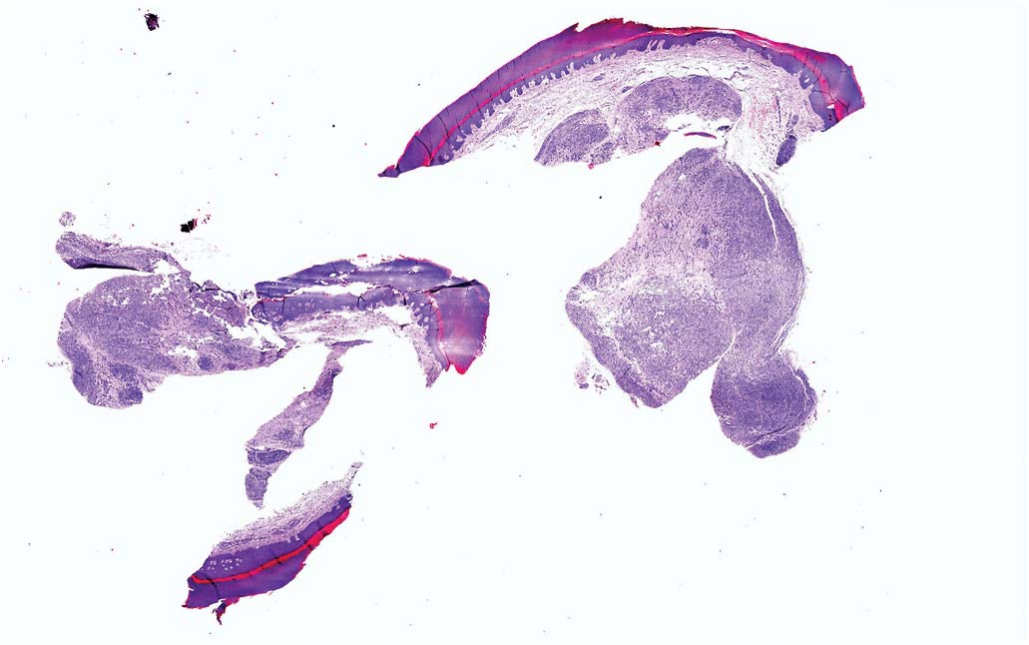
Microscopic low power view of dermal-based, well-circumscribed tumor, HE ×2.

**FIGURE 3.**
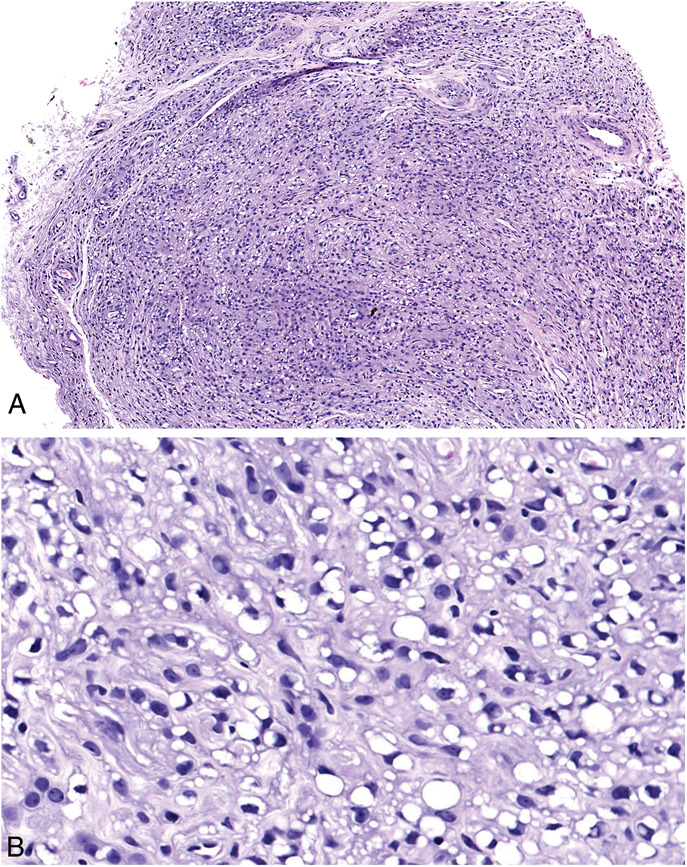
A, Diffuse intracytoplasmic vacuolization causing microreticular appearance, HE ×15. B, Pseudolipoblastic cells in a collagenous stroma, HE ×400.

Macroscopically, the lesion measured 0.5 cm in diameter with a nodular appearance (Fig. 1). Microscopically, low-power magnification revealed a normal-appearing acral epidermis and a well-defined but nonencapsulated nodular tumor in the dermis (Fig. 2). At higher magnifications, the tumor was composed of uniform, normal epithelioid cells with ovoid nuclei, arranged in a microreticular pattern in a collagenous stroma. The cells contained small, clear intracytoplasmic vacuoles that resembles signet ring cells and mimics the appearance of pseudolipoblast (Fig. 3A, B). Neither cytologic atypia nor mitosis was detected. There was no necrosis.

**FIGURE 4.**
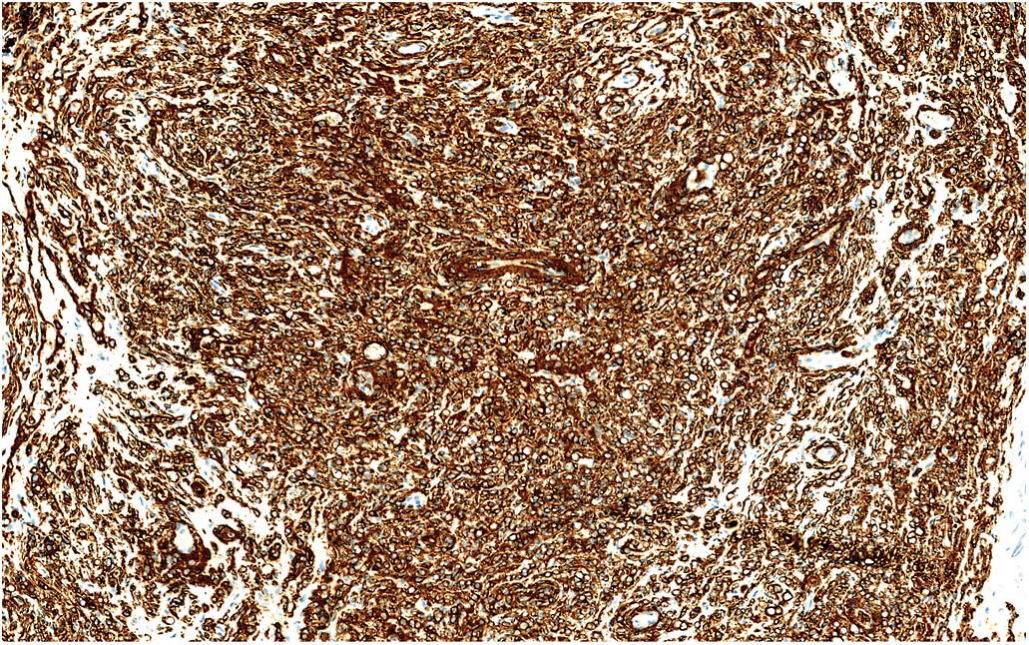
Immunohistochemical staining of tumor cells showing strong and diffusely positive Glut-1.

At immunohistochemistry, vimentin, EMA, and Glut-1 (Fig. 4) were diffusely positive. P63 and CK AE1/AE3 were stained with varying proportions and intensities. S-100, Sox-10, HMB-45, neurofilament, CD31, CD34, ERG, desmin, SMA, and calretinin were negative.

Histopathologic features and immunohistochemical profile, EMA and Glut-1 positivity and S-100 negativity in pseudolipoblastic cells, were consistent with the diagnosis of perineurioma.

## DISCUSSION

Benign peripheral nerve sheath tumors arise from the different cell types that are the components of nerve sheath. Perineurium is one of these components, forming a continuous structure with the pia-arachnoid of the central nervous system and, distally, with the sheath of capsular cells of peripheral sensory organs and proprioceptive receptors.^17^ Furthermore, apart from modulating external stretching forces, the perineurium also serves as the blood–nerve barrier. Perineuriomas are composed of perineurial cells with 2 histologic subtypes: intraneural perineuriomas and extraneural perineuriomas.^3^

Intraneural perineuriomas occur in children and young adults and were first described in 1964.^18^ It affects the major peripheral nerves and leads to neurologic symptoms such as loss of motor function or more rarely a sensory deficit.^19^ Histologically these tumors are characterized by proliferating perineurial cells that are organized into concentric layers around the central axon and Schwann cell, giving an “onion-bulb” appearance, especially in the sections that are transversal to the nerve.

The second subtype is extraneural perineuriomas. They arise in dermal subcutaneous, deep soft tissue, and are generally unassociated with peripheral nerves. One of the key histopathologic characteristics is the absence of residual Schwann cells or axons, which differ from intraneural perineuriomas. At microscopy, perineurioma typically shows a storiform to fascicular growth pattern, with lamellar arrangement, and perivascular whorls within a collagenous to myxoid stromal background. The tumor cells are slender, spindle shaped with wavy, tapering nuclei, and delicate bipolar cytoplasmatic processes.^1^

It is important to recognize different morphologic subtypes of perineuriomas to make correct diagnoses and to avoid unnecessary treatment. Sclerosing perineurioma is one of the most detected histologic subtypes of perineurioma, first defined by Tetsch and Miettinen in 1997.^20^ In their study, all lesions were located in the acral sites but the tumors are also defined in extra-acral/soft tissue.^21^ Pseudolipoblastic perineurioma is one of the rarest histologic subtypes and was described by Torres-Mora et al in 2016 in 2 cases. The cases were in the tongue and the triceps, and in both cases, the most prominent histologic feature was the presence of numerous small, clear vacuoles within the cells, which replaced and scalloped the nuclei, mimicking lipoblasts closely. When this vacuolization is particularly evident, it generates a “net-like” or microreticular appearance as seen in our case.^12^ It is also important to remind that peripheral nerve sheath tumors occasionally have adipocytic components.^22^ Although this is a very rare phenomenon in perineuriomas, the pathogenesis of having an adipose tissue component is not completely understood.^23^

Regardless of localization and morphologic subtypes, perineuriomas immunohistochemically are characterized by the expression of vimentin, EMA, Glut-1, and claudin-1. They are also frequently positive for CD34. S-100 is typically negative.^24^ EMA positivity and S-100 negativity help to rule out schwannomas that are S-100 positive and EMA negative.^3^

EMA (epithelial membrane antigen) is a transmembrane protein and, in general, it is useful for carcinomas with the other cytokeratin stains. Perineurial cells and perineuriomas show EMA positivity. It is also kept in mind that in a small subset of perineuriomas, EMA expression may be only focal and weak and also negative.^2,25^ Claudin-1 has also been described as a perineurial marker. It was first described in 1998 as a tight junction-associated protein 26, and it has been found in neoplastic and non-neoplastic perineural cells, whereas fibroblastic and myofibroblastic neoplasms are negative. Glut-1 (glucose transporter isoform 1) as a member of a family of glucose transporters is normally expressed in perineurial cells, erythrocytes, germinal centers of lymphoid tissue, renal tubules, and some carcinomas.^27^ Glut-1 is very useful for differential diagnosis of perineuriomas from schwannomas and neurofibromas that are Glut-1 negative. Glut-1 and claudin-1 are rather sensitive perineural markers that should be added to the immunohistochemical panel in cases where perineurioma is considered.^25^

Because of the predominance of vacuolated “pseudolipoblastic” cells, this lesion may raise suspicion of liposarcoma. Most cases of liposarcoma arise from deep soft tissue and pure dermal cases are exceedingly uncommon.^28^ Microscopically this tumor is characterized by pleomorphic lipoblasts that are S-100 positive and EMA negative. Therefore, considering the morphologic characteristics and immunohistochemical studies, it becomes evident that liposarcoma can be readily excluded. Pseudolipoblastic perineurioma should be differentiated from the tumors with vacuolated cells such as epithelioid hemangioendothelioma, signet ring cell carcinoma, or signet ring cell melanoma. In addition to clinicopathologic features, the immunohistochemical panel that contains antibodies for endothelial, epithelial, and melanocytic differentiation will be beneficial for the exact diagnosis.

In conclusion, it is important to remember perineuriomas in the differential diagnosis of many other mesenchymal tumors, especially in unusual localizations. Perineuriomas are benign tumors treated with simple excision. Being familiar with different morphologic subtypes of these tumors allows a correct diagnosis, avoiding unneccessary treatment.
